# Treatment of Subcortical Aphasia Due to Putaminal Hemorrhage With the Japanese Version of Melodic Intonation Therapy (MIT-J)

**DOI:** 10.7759/cureus.55590

**Published:** 2024-03-05

**Authors:** Midori Ueda, Koji Hayashi, Asuka Suzuki, Yuka Nakaya, Naoko Takaku, Toyoaki Miura, Mamiko Sato, Kouji Hayashi, Yasutaka Kobayashi

**Affiliations:** 1 Department of Rehabilitation Medicine, Fukui General Hospital, Fukui, JPN; 2 Graduate School of Health Science, Fukui Health Science University, Fukui, JPN

**Keywords:** non-fluent aphasia, motor aphasia, putaminal hemorrhage, neurological music therapy, melodic intonation therapy

## Abstract

Melodic intonation therapy (MIT) is one of the rehabilitation methods for patients with non-fluent or dysfluent aphasia, mainly caused by stroke or brain injury. Although MIT is conducted in various languages, reports on the Japanese version of MIT (MIT-J) are limited. In this report, we describe a case about the efficacy of MIT-J in the subacute phase after stroke on subcortical aphasia. Our case was a 60-year-old right-handed woman who suffered from left putaminal hemorrhage. She was treated with acute therapy, including medications and rehabilitation, but non-fluent aphasia was preserved. Regardless of general speech therapies, her aphasia was not improved. In the subacute phase, we started MIT-J (protocol: 20 minutes per day, five days per week for two weeks). The effect of MIT-J was remarkable and in particular, speech intelligibility was improved. It is required to accumulate more cases to reveal the effect of MIT-J.

## Introduction

Melodic intonation therapy (MIT) is an intonation-based therapeutic method for patients with non-fluent or dysfluent aphasia, mainly caused by stroke or brain injury [1,2]. It was developed in response to the observation that patients with severe aphasia are often able to produce clear, linguistically accurate words while singing, but not during speech [3,4]. MIT is one of the effective methods for aphasia validated by the American Academy of Neurology (AAN) [5]. The original version of MIT has been adapted to different clinical populations, including non-English language populations such as Romanian, Persian, Italian, and Japanese, with comparable clinical results [1]. The Japanese version of MIT (MIT-J) was developed in the 1980s [6]. However, reports about the efficiency of MIT-J are still limited [2], and its effectiveness or adaptation has not been sufficiently investigated. In addition, as far as we know, limited reports are published about the effectiveness of music therapy including MIT for aphasia due to cerebral hemorrhage including putaminal hemorrhage [2,7]. We describe a report about the efficacy of MIT-J on subcortical aphasia by putaminal hemorrhage in the subacute phase after stroke.

## Case presentation

**Figure 1:**
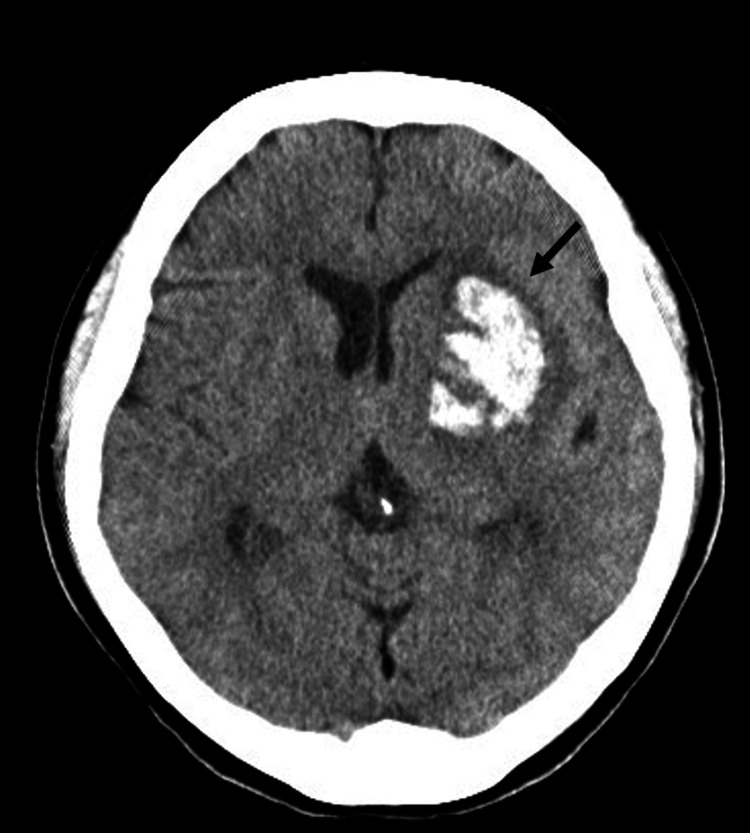
Brain computed tomography (CT) on admission. Brain CT on admission showed a hematoma in the left putamen (arrowhead). The volume of the hematoma was estimated to be 31 mL.

A 60-year-old right-handed woman, who had a medical history of hypertension, lumbar disc herniation, and bilateral osteoarthritis, developed disturbed consciousness. Her family history was unremarkable. She was transported to a hospital where a neurological examination revealed disturbed consciousness, motor aphasia, and right hemiparalysis. Her National Institutes of Health Stroke Scale (NIHSS) score was evaluated as 17 points. A brain CT showed left putaminal hemorrhage (Figure 1).

**Figure 2:**
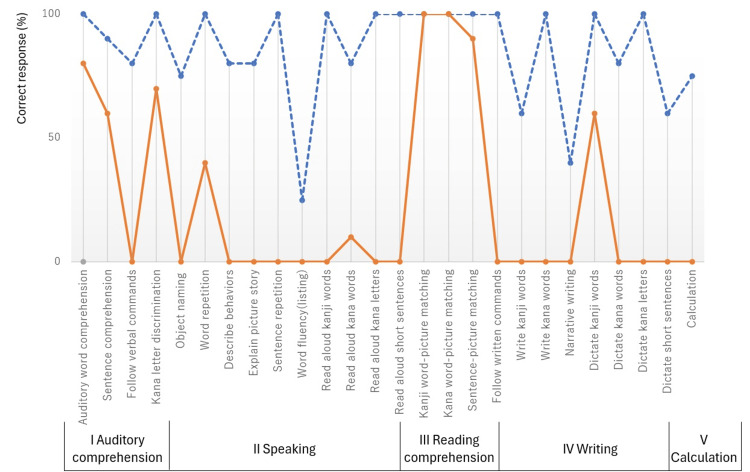
Profile of the Standard Language Test of Aphasia (SLTA). The results of SLTA before and after MIT-J. Solid line; our patient’s score before MIT-J. Broken line; score after MIT-J.

The volume of the hematoma was estimated to be 31 ml. She was treated with antihypertensive medications and hemostatic agents intravenously. Subsequently, amlodipine 5 mg was prescribed. After acute treatment, she was transferred to our hospital for rehabilitation therapy on Day 21. Neurological examination on admission showed good language understanding for short sentences, disturbed language expression, and hemiplegia (Brunnstrom stage; the upper extremity: III, the fingers: II, the lower extremity: IV). Profile of the Standard Language Test of Aphasia (SLTA) on admission revealed that she had extreme difficulty with naming, reading aloud, and writing, and was diagnosed with non-fluent aphasia (Figure 2, solid line). On Day 28, language understanding and repetition failure were improved, but phonemic paraphasia and monotone voice were noted. A diagnosis of subcortical aphasia was made which did not improve for 2-months after admission. We started MIT-J (protocol: 20-minute per day, five days per week, two weeks) on Day 61 along with other speech therapy. MIT-J was treated by a speech therapist who had a license for MIT. The target words were selected according to the severity of aphasia and the need in her daily life. The patient completed all MIT programs while hospitalized. After intervention by MIT-J, a reduction of phonological errors, an increase in self-correction, and improvement of rhythm and intonation were noted. Additionally, her SLTA was significantly improved in diffuse profiles (Figure 2, dash line). Most importantly, her speech intelligibility was improved. She was discharged from our hospital on Day 120.

## Discussion

This report describes the efficiency of MIT-J on subcortical aphasia by putaminal hemorrhage in the subacute phase of a stroke recovery process. The subcortical aphasia was due to a relatively large-sized putaminal hemorrhage, with a capacity estimated at 31 ml. Her aphasia was diagnosed as non-fluent aphasia. Although the patient was not fully improved by other speech therapy, MIT-J was effective for the aphasia. In particular, speech intelligibility was improved.

Regarding the treatment of non-fluent aphasia, music therapies have been one option, because anecdotal and experimental studies have shown that patients with Broca’s aphasia have the ability to sing [8,9]. Among music therapies, MIT is a common rehabilitation program for speech production disorders including non-fluent aphasia [9]. The effectiveness of MIT has been reported overseas, but there are limited reports from Japan. We applied MIT-J to our patient according the method described in a previous report [1]. MIT-J has two major different points from the original English version because the phonological unit in the Japanese language is a mora, a temporal unit that divides words into almost isochronous segments [1]. First, the MIT-J uses two pitches, high and low, whereas the original MIT has several pitches. Second, in MIT-J, there are two moras in a single beat of left-hand tapping [1]. These modifications may make MIT-J easier and more effective for broader aphasic symptoms than the original [1].

The mechanisms of the usefulness of music therapy for patients with non-fluent aphasia have been considered in the previous reports. It has been reported that the right hemisphere region becomes more active during singing [2, 10-12]. It is thought that music therapy, including melodic elements, is one of the potential therapies for non-fluent aphasia because singing can activate the right hemisphere in patients and may compensate for a left hemisphere lesion [13]. It is reported that MIT also activates the right hemisphere, in particular the central anterior gyrus, central anterior sulcus, central posterior gyrus, middle frontal gyrus, superior temporal gyrus, superior temporal sulcus, middle temporal gyrus, inferior temporal gyrus, lingual gyrus, and angular gyrus, in patients with aphasia [1,2]. These areas include Broca's area, the auditory cortex, and the other cortex responsible for their connection [2]. Contralateral hemisphere activation by MIT affects the white matter structure of the auditory-motor neural circuit compensation to promote the ability to encode and integrate verbal information [2]. This trans-hemisphere “mirror effect” has an important mechanism in the language recovery of patients with aphasia [2]. Additionally, regarding the evidence of a direct effect of MIT on aphasia, the absolute number of fibers in the right arcuate fasciculus was increased compared to before MIT as detected by diffusion tensor imaging [14]. Thus, MIT may induce functional and structural changes in a right hemisphere fronto-temporal network [14].

In our case, our patient had severe phonological distortion despite treatment with speech therapy. Furthermore, it has been reported that the prognosis of speech disorders due to putaminal hemorrhage worsens depending on the size of the hematoma [15]. In particular, the prognosis is poorer in patients with over 12 cm^2^ of hematoma [15]. Because the hematoma size in our case was relatively large, it was assumed that the prognosis for speech may be poor. After MIT-J, the SLTA was significantly improved in almost all profiles. Additionally, speech intelligibility was improved. Therefore, we believe that the effects of MIT-J are remarkable and effective.

This report has three major limitations. First is a single report. Therefore, the usefulness of MIT-J cannot be generalized immediately. Second, this study was conducted in the subacute phase of putaminal hemorrhage. Therefore, we cannot exclude the natural course of brain recovery after stroke even though the patient's aphagia was resolved after MIT-J. In the future, studies are needed in the chronic phase. Third is the timing of the SLTA evaluation. We evaluated SLTA two times; on Day 21, and after MIT-J treatment. Therefore, the reasons for improvement in SLTA may also include other speech therapies and the natural course. If the pure effect of MIT-J was to be evaluated, SLTA should have been evaluated immediately before MIT-J intervention. More studies are required to demonstrate a clear role of MIT-J in the treatment of post-stroke aphasia.

## Conclusions

We reported a case of subcortical and non-fluent aphasia treated with MIT-J. To date, the effectiveness of various music therapies for post-stroke aphasia has been reported, but there have been few reports on MIT-J in the treatment of post-stroke aphasia. We believe that MIT-J can be a useful option as a rehabilitation therapy in similar cases. More studies are required to demonstrate a clear role of MIT-J in the treatment of post-stroke aphasia.
